# Optimization of extraction-free protocols for SARS-CoV-2 detection using a commercial rRT-PCR assay

**DOI:** 10.1038/s41598-023-47645-0

**Published:** 2023-11-21

**Authors:** Minhee Kang, Eunjung Jeong, Ji-Yeon Kim, Sun Ae Yun, Mi-Ae Jang, Ja-Hyun Jang, Tae Yeul Kim, Hee Jae Huh, Nam Yong Lee

**Affiliations:** 1 Smart Healthcare Research Institute, Biomedical Engineering Research Center, Samsung Medical Center, Seoul, South Korea; 2 Department of Medical Device Management and Research, Samsung Advanced Institute for Health Sciences & Technology, Sungkyunkwan University, Seoul, South Korea; 3 Samsung Biomedical Research Institute, Center for Clinical Medicine, Samsung Medical Center, Seoul, South Korea; 4 Department of Laboratory Medicine and Genetics, Samsung Medical Center, Sungkyunkwan University School of Medicine, Seoul, South Korea

**Keywords:** Molecular biology, Infectious-disease diagnostics, Biomedical engineering

## Abstract

In the ongoing global fight against coronavirus disease 2019 (COVID-19), the sample preparation process for real-time reverse transcription polymerase chain reaction (rRT-PCR) faces challenges due to time-consuming steps, labor-intensive procedures, contamination risks, resource demands, and environmental implications. However, optimized strategies for sample preparation have been poorly investigated, and the combination of RNase inhibitors and Proteinase K has been rarely considered. Hence, we investigated combinations of several extraction-free protocols incorporating heat treatment, sample dilution, and Proteinase K and RNase inhibitors, and validated the effectiveness using 120 SARS-CoV-2 positive and 62 negative clinical samples. Combining sample dilution and heat treatment with Proteinase K and RNase inhibitors addition exhibited the highest sensitivity (84.26%) with a mean increase in cycle threshold (Ct) value of + 3.8. Meanwhile, combined sample dilution and heat treatment exhibited a sensitivity of 79.63%, accounting for a 38% increase compared to heat treatment alone. Our findings highlight that the incorporation of Proteinase K and RNase inhibitors with sample dilution and heat treatment contributed only marginally to the improvement without yielding statistically significant differences. Sample dilution significantly impacts SARS-CoV-2 detection, and sample conditions play a crucial role in the efficiency of extraction-free methods. Our findings may provide insights for streamlining diagnostic testing, enhancing its accessibility, cost-effectiveness, and sustainability.

## Introduction

The Coronavirus disease 2019 (COVID-19) has brought considerable challenges to the global public health system, as well as notable environmental consequences due to increased plastic usage associated with diagnostic procedures^1–4^. While the COVID-19 pandemic triggered remarkable progress in the field of molecular diagnostics, particularly in the context of detecting severe acute respiratory syndrome coronavirus 2 (SARS-CoV-2) detection, the sample preparation for real-time reverse transcription polymerase chain reaction (rRT-PCR) continues to be a bottleneck in the overall diagnostic testing workflow^5–9^. The preparation process typically involves extracting and purifying viral RNA from patient samples, which introduces additional steps and time into the testing process. This step is critical for eliminating potential inhibitors and ensuring reliable results. Various commercial kits and methods are available for RNA extraction, and laboratories and diagnostic centers have developed protocols to perform this procedure efficiently^10–12^. However, manual extraction methods used in many laboratories can be time-consuming and require skilled technicians for accurate execution. In addition, the use of plastic materials associated with RNA extraction kits and reagents contributes to environmental concerns and escalates the overall cost of diagnostic testing^13–15^. To tackle this issue, efforts have been made to address bottlenecks and streamline sample preparation. Automated and high-throughput systems have been developed to increase efficiency and reduce the labor required for RNA extraction. These systems can simultaneously process multiple samples and may incorporate robotic platforms or magnetic bead-based technologies to improve throughput^16–19^. Furthermore, researchers are exploring alternative methods of RNA extraction that are more cost-effective and less labor-intensive. Some of these methods include the use of magnetic beads^20,21^, solid-phase extraction^22,23^, and microfluidic devices^24–26^, which simplify the extraction process and reduce the associated costs.

In addition to technical advancements, initiatives have been made to optimize protocols and develop extraction-free methods^27–32^. Extraction-free rRT-PCR methods aim to simplify and expedite the testing process by bypassing the RNA extraction step, which is time-consuming, labor-intensive. These methods often involve the direct addition of specific reagents or buffers to the patient sample to inactivate potential inhibitors and facilitate the direct amplification of viral RNA. Additionally, one of the practical advantages is their cost-effectiveness compared to extraction kit. For example, the average cost incurred per sample using extraction kits falls within the range of 6–12 USD, while the cost associated with the application of specific reagents such as Proteinase K and RNase inhibitors in the conducted experiments amounts to merely 1.15 USD per sample^33^. The performance of extraction-free rRT-PCR methods can vary depending on the protocol and patient sample characteristics^34–36^.

The most straightforward extraction-free method is to dilute the sample to reduce inhibitory substances that might interfere with the PCR. While the optimal dilution for extraction-free methods may vary depending on the specific assay and sample type, several studies have reported successful detection using a 1:1 dilution^33,37,38^. Other extraction-free methods, such as thermal or direct lysis, may also be used in combination with or as alternatives to dilution^32,39–41^. These methods involve the disruption of cell membranes through heat, allowing nucleic acid molecules to be accessible for PCR amplification without the need for a separate extraction step. The impact on reducing the occurrence of invalid results and minimizing the risk of contamination in unextracted rRT-PCR through the utilization of Proteinase K and RNase inhibitors has also been reported^28,33,42^. The summary of extraction-free protocols and their corresponding sensitivity in previous studies can be found in Table S1 in the supplementary information. However, there has been a lack of study on optimized strategies that combine sample dilution with Proteinase K and RNase inhibitors along with heat treatment. Validation and evaluation studies are necessary to ensure that these methods yield reliable and accurate results, aligning with the performance standards of traditional rRT-PCR, especially when using samples having diverse viral concentrations.

Herein, we validated the use of heat treatment and sample dilution as extraction-free methods for detecting SARS-CoV-2. Proteins can interfere with the extraction process, and the use of Proteinase K improves viral nucleic acid accessibility by dissolving sample proteins^33,42,43^. Ribonucleases are enzymes that can degrade RNA, and the use of RNase inhibitors helps inhibit their activity, preserving the integrity of viral RNA during sample preparation. Therefore, we also incorporated Proteinase K and RNase inhibitors for improving viral nucleic acid detection. Combining methods, including heat treatment, sample dilution, Proteinase K, and RNase inhibitors, we aimed to develop an optimal extraction-free method for viral nucleic acid detection. This approach simplifies the sample preparation process and may improve the efficiency and scalability of diagnostic testing, particularly during situations like a pandemic, where large-scale testing is required. By streamlining the RNA extraction process, laboratories can enhance their testing capacities, reduce turnaround times, and make diagnostic processes more accessible and cost-effective.

## Result and discussion

The efficiency of each extraction-free protocol was evaluated by comparing differences in Ct values (cycle threshold) with those obtained using the standard methodology using the extraction step (Group I). The experimental groups consisted of variations in the extraction-free protocols, denoted as Groups II–VI. Specifically, the groups were categorized as follows: Group I (standard protocol), Group II (extraction-free protocol based on heat treatment), Group III (extraction-free protocol based on heat treatment and sample dilution), Group IV (extraction-free protocol based on heat treatment, sample dilution, and addition of Proteinase K), Group V (extraction-free protocol based on heat treatment, sample dilution, and addition of RNase inhibitors), and Group VI (extraction-free protocol based on heat treatment, sample dilution, and addition of Proteinase K and RNase inhibitors). Detailed information can be found in the “Methods” section and Table S2 of the supplementary information. Overall, 170 samples—108 positives and 62 negatives—were included in our evaluation with the standard methodology using the extraction step recommended by the manufacturer. Among positive samples, 46 (46/108, 42.6%) showed Ct values < 20, 36 (36/108, 33.3%) had intermediate Ct values (20 ≤ Ct ≤ 30), and 26 (26/108, 24.1%) showed low Ct values (Ct > 30) for the ORF1ab gene. Similarly, for the N gene, 54 (54/108, 50%) showed Ct values < 20, 34 (34/108, 31.5%) had intermediate Ct values (20 ≤ Ct ≤ 30), and 20 (20/108, 18.5%) showed low Ct values (Ct > 30). The Ct values obtained from the experimental groups using different extraction-free protocols correlated with those obtained with the standard methodology (Fig. S1 in the supplementary information). However, false negatives were also observed for each protocol.

**Figure 1:**
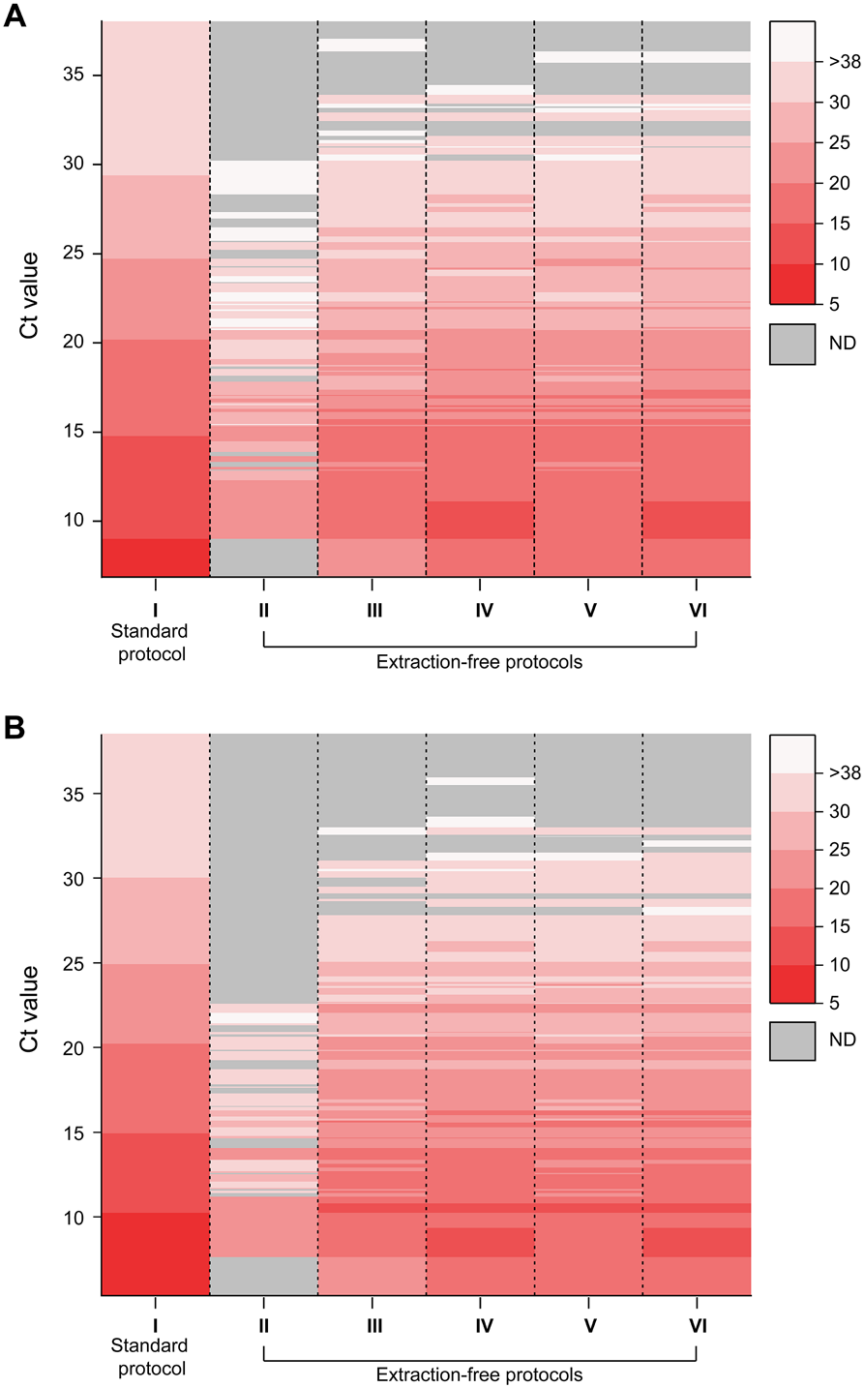
Heat map of Ct values for (**A**) the ORF1ab gene and (**B**) the N gene subjected to various experimental conditions compared to a standard methodology using the extraction step. Gray color represents lower Ct values of samples labeled as not detected. (Ct values determined by I, standard methodology using the extraction step; II, extraction-free protocol based on heat treatment; III, extraction-free protocol based on heat treatment and sample dilution; IV, extraction-free protocol based on heat treatment, sample dilution, and addition of Proteinase K; V, extraction-free protocol based on heat treatment, sample dilution, and addition of RNase inhibitors; VI, extraction-free protocol based on heat treatment, sample dilution, and addition of Proteinase K and RNase inhibitors).

**Figure 2:**
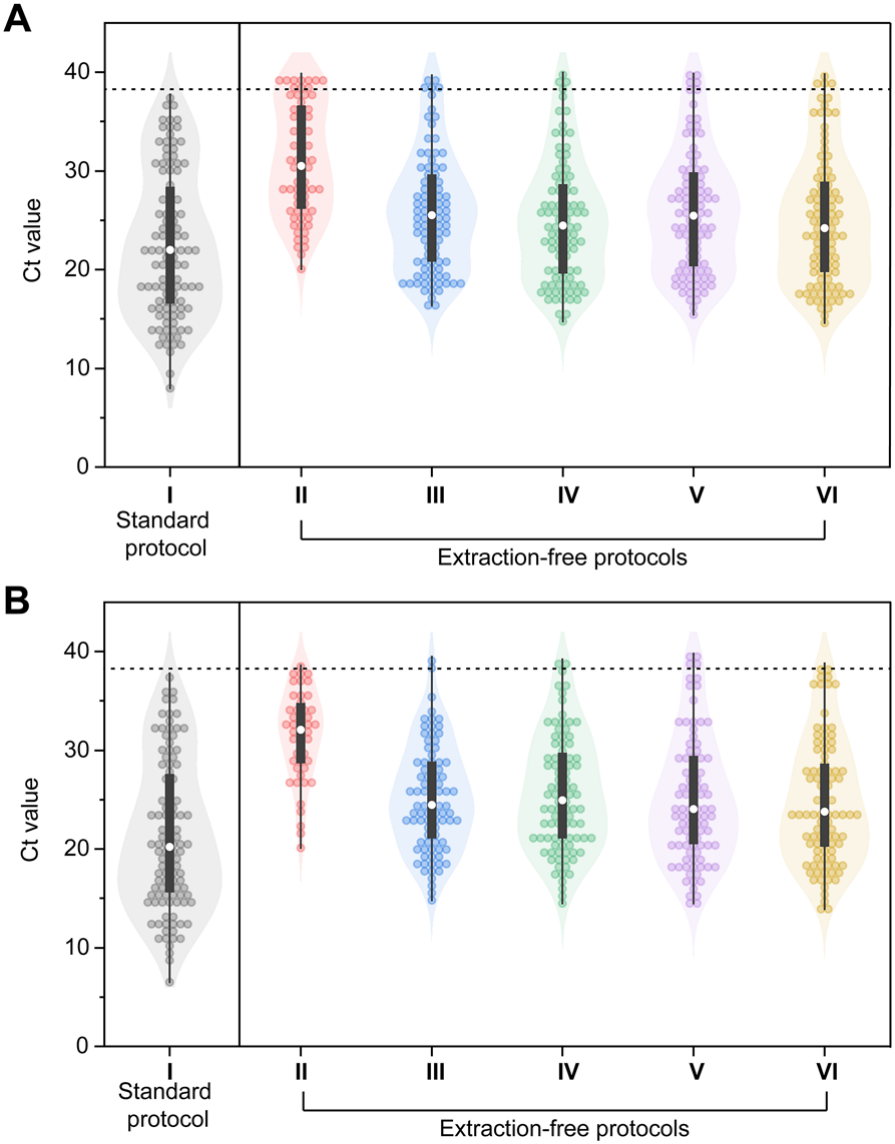
The individual and distribution of Ct values of various experimental conditions for (**A**) the ORF1ab gene and (**B**) the N gene. The median of each group is presented as the white circle with an interquartile range box and range. Dotted lines show the positive thresholds for ORF1ab and N genes. (Ct values determined by I, standard methodology using the extraction step; II, extraction-free protocol based on heat treatment; III, extraction-free protocol based on heat treatment and sample dilution; IV, extraction-free protocol based on heat treatment, sample dilution, and addition of Proteinase K; V, extraction-free protocol based on heat treatment, sample dilution, and addition of RNase inhibitors; VI, extraction-free protocol based on heat treatment, sample dilution, and addition of Proteinase K and RNase inhibitors).

Figure 1 comprehensively summarizes the Ct value distributions across experimental groups. In Group II, regardless of the target genes, no targets were detected for samples with Ct values above 30 (Fig. 1A). The median in the Ct values for the ORF1ab and N gene varied across experimental conditions: Group II (30.48, 32.11), Group III (25.53, 24.49), Group IV (24.44, 24.03), Group V (25.49, 24.77), and Group VI (24.14 23.77). These findings indicate that sample dilution notably affected nucleic acid detection. Other factors, namely, treatment with Proteinase K and RNase inhibitors contributed to only a marginal improvement compared with Group III, in which samples were treated with a combination of dilution and heating and did not yield statistically significant differences in Ct values (Fig. 2). Bland–Altman plots were used to compare the extraction-free protocols with the standard methodology using the extraction step (Fig. S2 in supplementary information). The differences in the Ct values across extraction-free protocols compared with the standard protocol are shown in Table 1. In the context of sample enrichment and purification, the typical nucleic acid extraction step can lead to a four-fold increase in sample concentration, wherein 200 µL of the sample is concentrated to 50 µL of eluate. During the extraction process, various inhibitory substances can be co-purified along with RNA, potentially interfering with downstream PCR. In contrast, the extraction-free protocol involves a 1:1 dilution of the sample, resulting in a two-fold dilution or 0.5 times the concentration of the original sample. As a result, a significant eight-fold variation in amplification results may be observed when comparing identical samples. Considering the logarithmic scale of the Ct values of quantitative PCR, a mere ten-fold difference between samples corresponds to a 3.3 Ct difference under the assumption of 100% PCR efficiency. Interestingly, in our study, Group VI exhibited a mean increase in Ct value of + 3.8 compared to Group I, supporting the expected variation in results.

**Table 1:** Ct values compared to those observed with the standard reference for ORF1ab gene.

	Systematic differences			Regression
	Mean	Standard deviation (SD)	95% CI	P
I–II	− 12.2	3.2	− 12.93 to 11.39	< 0.0001
I–III	− 4.9	2.0	− 5.34 to 4.52	0.2614
I–IV	− 4.1	1.6	− 4.38 to 3.74	0.7449
I–V	− 4.7	1.8	− 5.10 to 4.37	0.4243
I–VI	− 3.8	1.5	− 4.07 to 3.46	0.8974

I, standard methodology using the extraction step; II, extraction-free protocol based on heat treatment; III, extraction-free protocol based on heat treatment and sample dilution; IV, extraction-free protocol based on heat treatment, sample dilution, and addition of Proteinase K; V, extraction-free protocol based on heat treatment, sample dilution, and addition of RNase inhibitors; VI, extraction-free protocol based on heat treatment, sample dilution, and addition of Proteinase K and RNase inhibitors.

**Figure 3:**
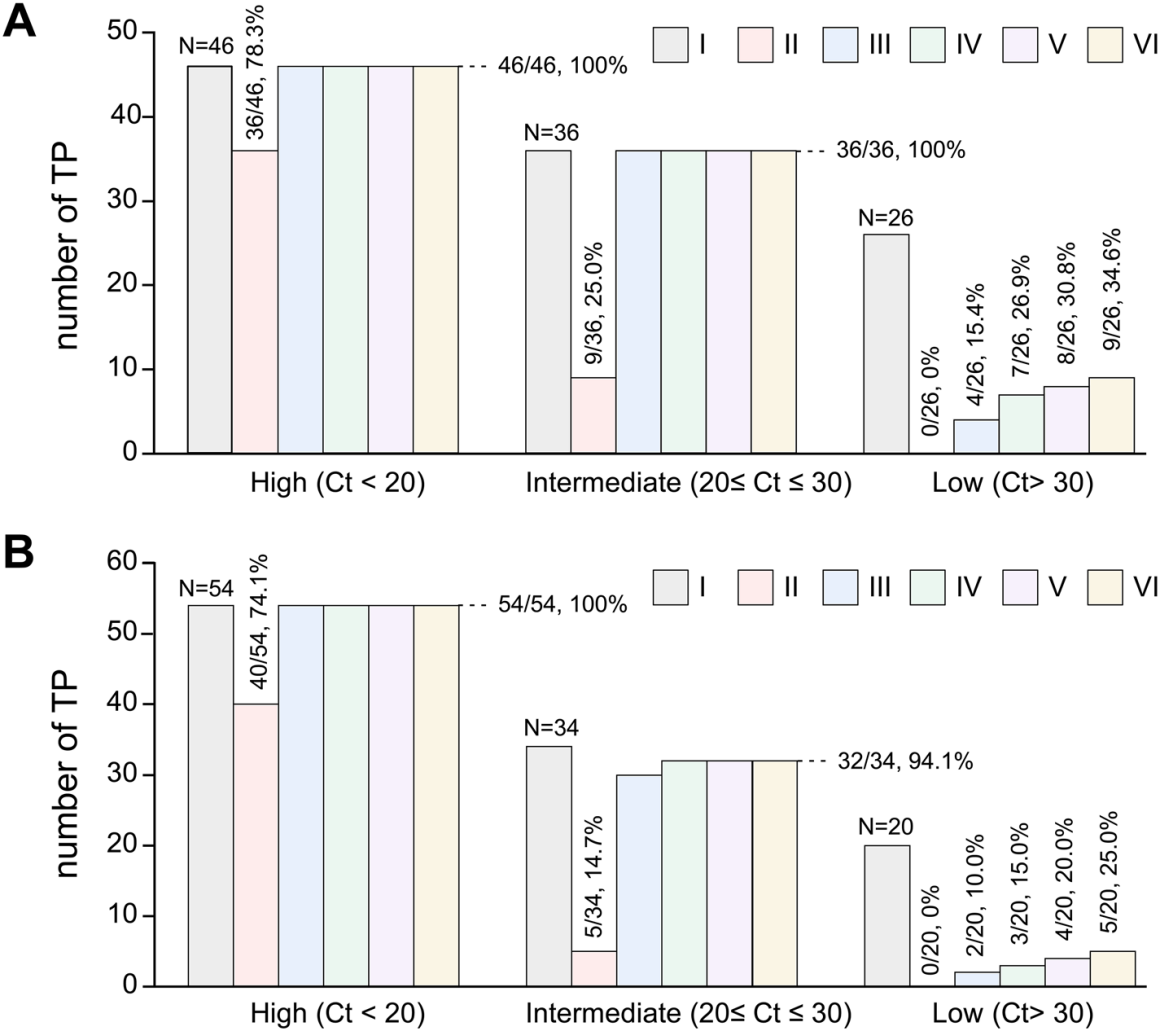
True positive fractionation across all experimental conditions for (**A**) the ORF1ab gene and (**B**) the N gene. SARS-CoV-2 RNA loads were categorized as high (Ct values less than 20), intermediate (Ct values of 20–30), or low (Ct values of more than 30) determined by standard methodology using the extraction step (Group I). I, standard methodology using the extraction step; II, extraction-free protocol based on heat treatment; III, extraction-free protocol based on heat treatment and sample dilution; IV, extraction-free protocol based on heat treatment, sample dilution, and addition of Proteinase K; V, extraction-free protocol based on heat treatment, sample dilution, and addition of RNase inhibitors; VI, extraction-free protocol based on heat treatment, sample dilution, and addition of Proteinase K and RNase inhibitors.

The detection rates based on Ct values for ORF1ab are summarized in Fig. 3. In the extraction-free protocols, lower detection rates were observed for samples with Ct values above 30 for the ORF1ab gene: Group II (0/26, 0%), Group III (4/26, 15.4%), Group IV (7/26, 26.9%), Group V (8/26, 30.8%), and Group VI (9/26, 34.6%). Similarly, lower detection rates were observed for samples with Ct values above 30 for the N gene: Group II (0/20, 0%), Group III (2/20, 10.0%), Group IV (3/20, 15.0%), Group V (4/20, 20.0%), and Group VI (5/20, 25.0%).

The sensitivity and specificity in each experimental group are shown in Table 2. Although the sensitivity of all experimental conditions was notably lower than that of the standard methodology, we observed substantial improvements among different conditions in response to sample dilution. Moreover, when combined with heat treatment, sample dilution resulted in a sensitivity of 79.63%, which represents a marked improvement of 38% compared with the application of heat treatment alone. In addition, the incorporation of Proteinase K and RNase inhibitors in conjunction with sample dilution and heat treatment contributed to a further slight improvement in sensitivity, which can be attributed to a marginal improvement in the low Ct group (Ct > 30). Although the inclusion of Proteinase K and RNase inhibitors has shown benefits in numerous studies, it is imperative to consider certain factors when implementing them in extraction-free PCR methods. First, the concentration and incubation time of these reagents should be optimized to ensure their effectiveness without causing adverse effects on the PCR. Second, the compatibility of these reagents under different sample conditions should be carefully evaluated to ensure consistent and reliable performance. Indeed, our experimental findings clearly showed a consistent occurrence of false negatives in a number of samples across extraction-free protocols that involved heat treatment and sample dilution (Table S3 in the supplementary information). Because the efficiency of extraction-free PCR can be significantly influenced by the condition of the sample, factors such as sample quality, presence of inhibitors, and composition of the sample matrix should be factored in when implementing extraction-free PCR methods to develop strategies for maximizing the accuracy and reliability of this approach. Finally, the cost-effectiveness and scalability of incorporating Proteinase K and RNase inhibitors should be considered, as they may contribute to the overall expenses of diagnostic testing.

**Table 2:** Comparison of diagnostic performance across all experimental conditions.

	TP	FP	TN	FN	Sensitivity (95% CI)	Specificity (95% CI)
II	45	0	62	63	41.67 (32.25–51.55)	100 (94.22–100)
III	86	0	62	22	79.63 (70.80–86.77)	100 (94.22–100)
IV	89	0	62	19	82.41 (73.90–89.)	100 (94.22–100)
V	90	0	62	18	83.33 (74.94–89.81)	100 (94.22–100)
VI	91	0	62	17	84.26 (76.00–90.55)	100 (94.22–100)

II, extraction-free protocol based on heat treatment; III, extraction-free protocol based on heat treatment and sample dilution; IV, extraction-free protocol based on heat treatment, sample dilution, and addition of Proteinase K; V, extraction-free protocol based on heat treatment, sample dilution, and addition of RNase inhibitors; VI, extraction-free protocol based on heat treatment, sample dilution, and addition of Proteinase K and RNase inhibitors.

In this study, we aimed to optimize the extraction-free approach, simplify the sample preparation process, and potentially increase testing efficiency and scalability. To this end, we validated the use of extraction-free protocol based on heat treatment and sample dilution while incorporating Proteinase K and RNase inhibitors to enhance nucleic acid extraction efficiency. Our results demonstrate that Group VI, which included all parameters (extraction-free protocols based on heat treatment, sample dilution, and addition of Proteinase K and RNase inhibitors), consistently showed the lowest average Ct values for all target genes. In contrast, Group II, which involved only heat treatment protocol, had the highest average Ct value. Importantly, most false negatives observed using the extraction-free protocols incorporating sample dilution exhibited Ct values above 30, regardless of the target gene. However, the extraction-free protocol based solely on heat treatment exhibited lower detection rates even for samples with a Ct value of less than 30 in the standard protocol. Our findings highlight the significant influence of sample conditions on the efficiency of extraction-free methods. Sample quality, presence of inhibitors, and composition of the sample matrix are among the factors that can remarkably affect amplification efficiency. However, we found that it is crucial to further validate these methods to ensure their reliability and comparability, particularly in the context of massive sample testing. Furthermore, additional prospective studies are required to substantiate the effect of sample conditions on the efficiency of extraction-free methods. We believe the findings of this study contribute to the ongoing efforts to streamline diagnostic testing, improve efficiency, enhancing its accessibility, cost-effectiveness, and sustainability by minimizing plastic waste and chemical reagent usage, especially in large-scale testing scenarios such as pandemics.

## Conclusion

In conclusion, this study emphasizes the potential of extraction-free methods for SARS-CoV-2 detection. By optimizing the sample preparation process and incorporating specific treatments and reagents, we demonstrated the feasibility of an extraction-free approach for enhancing nucleic acid detection. Our study revealed that sample dilution had a notable impact on the testing process, whereas the influence of Proteinase K and RNase inhibitors was found to be insignificant. Additional large-scale prospective studies are required to substantiate the effect of sample conditions on the efficiency of extraction-free methods.

## Methods

### Ethics statement

The study protocol was reviewed and approved by the Institutional Review Board of Samsung Medical Center, Seoul, Republic of Korea (IRB No. SMC 2023-03-031) and conducted in accordance with relevant guidelines and regulations. The requirement for informed consent was waived by the Institutional Review Board of Samsung Medical Center because of the retrospective nature of the study and the use of anonymized patient data.

### Preparation of clinical specimens

A total of 182 respiratory SARS-CoV-2 nasopharyngeal swab (NPS) specimens (120 positive and 62 negative samples) collected in a viral transport medium (VTM; Noble Bioscience, Inc., Hwaseong, Republic of Korea) were de-identified to ensure patient anonymity. Each sample was aliquoted into individual microcentrifuge tubes based on the required volume for RNA extraction and extraction-free methods and stored at − 80 °C until further experiments. All sample preparation, processing, and PCR settings were performed within a class 2 Biosafety Cabinet located in a Biosafety Level 2 (BSL2) facility.

### RNA extraction

Nucleic acid extraction was conducted using the MagMax viral/pathogen nucleic acid isolation kit and the KingFisher Flex purification system (Thermo Fisher Scientific Inc., Massachusetts, United States) following the manufacturer’s instructions. Briefly, 200 μL of each sample was mixed with 530 μL of a binding solution containing an appropriate concentration of guanidine thiocyanate for effective virus inactivation. This mixture was then supplemented with 20 μL of magnetic beads and 10 μL of Proteinase K to increase the extraction yield. Subsequently, the purification system automatically carried out the subsequent steps, including washing, elution, and tip comb plate preparation.

### Real-time reverse transcriptase-polymerase chain reaction

Direct clinical specimens treated with the extraction-free protocols and extracted nucleic acids from clinical specimens served as inputs for real-time reverse transcriptase-polymerase chain reaction (rRT-PCR). The STANDARD™ M SARS-CoV-2 (SD BIOSENSOR, Suwon, Republic of Korea) was used for the detection of SARS-CoV-2 RNA, specifically targeting the N (nucleocapsid) and ORF1ab (polyprotein) genes, along with RNaseP (ribonuclease P) as an internal control, in clinical samples, following the manufacturer’s instructions. rRT-PCR was conducted using a CFX96 Real-Time System (Bio-Rad, Hercules, CA, United States). The thermal cycling steps consisted of reverse transcription at 50 °C for 5 min, initial denaturation at 95 °C for 3 min, pre-amplification with 5 cycles at 95 °C for 1 s and 60 °C for 1 s, followed by amplification with 40 cycles at 95 °C for 1 s and 60 °C for 1 s. Positive test results were determined based on the simultaneous detection of both SARS-CoV-2 target genes in accordance with the established guidelines provided by the World Health Organization (WHO). Of the 182 samples, 12 were excluded because of RNA degradation. This decision was made based on discrepancies between the routinely tested results upon arrival at the laboratory and the standard PCR results obtained in this study.

### Extraction-free protocols

With the exception of the protocol performed with heat treatment alone, all samples underwent an optimal dilution of 1:1. Additionally, two variations were explored: thermal lysis alone and thermal lysis combined with the addition of RNase inhibitors (Catalog # N8080119, Thermo Fisher Scientific Inc., Massachusetts, United States) and Proteinase K (Catalog # MC5005, Roche Diagnostics GmbH, Mannheim, Germany). For experimental groups II–VI, the samples were heated to 98 °C for 5 min, followed by rapid cooling at 4 °C before the rRT-PCR process. In Groups IV and VI, Proteinase K was directly added to RNase-free water before dilution at a final concentration of 100 μg/mL. Additionally, for this group, a 15-min heat treatment at 55 °C was performed before heating the sample at 98 °C for 5 min. Group V and VI underwent treatment with 1U/μL of RNase inhibitors as per the manufacturer’s protocol.

### Statistical analysis

The cycle threshold (Ct) was determined automatically using CFX Manager Software version 3.1 (Bio-Rad). Results were interpreted as positive only if the Ct values obtained for both ORF1ab and N were within the cutoff value, in accordance with the manufacturer's recommendations. The data obtained were described in terms of categorical variables divided into high (Ct values less than 20), intermediate (Ct values between 20 and 30), or low (Ct values greater than 30) categories. The sensitivities and specificities of each condition were assessed based on the results obtained when including the extraction step as the reference standard methodology. An independent *t*-test was used for the comparison of Ct values between groups, and McNemar’s test was used to compare diagnostic accuracy. A *p*-value of < 0.05 was considered to be statistically significant. Bland–Altman analysis was used to determine the degree of agreement based on the mean difference and standard deviation (SD) of the positive results.

### Supplementary Information

Supplementary Information.

## Data Availability

The data that support the findings of this study will be made available from the corresponding author on reasonable request.
