# Supplementary material for: Optimization of extraction-free protocols for SARS-CoV-2 detection using a commercial rRT-PCR assay
Source: Sci Rep. 2023 Nov 21;13:20364. doi: 10.1038/s41598-023-47645-0 (PMC10663557; doi:10.1038/s41598-023-47645-0)
Supplement: Supplementary file 1 — Supplementary Information. [file 41598_2023_47645_MOESM1_ESM.pdf]

Supplementary Information

Optimization of Extraction-Free Protocols for SARS-CoV-2 Detection using a Commercial rRT-PCR Assay

Minhee Kang[1,2,¶], Eunjung Jeong[1,2,¶], Ji-Yeon Kim[3], Sun Ae Yun[3], Mi-Ae Jang[4], Ja-Hyun Jang[4], Tae Yeul Kim[4,*], Hee Jae Huh[4,5,*], Nam Yong Lee[4]

[1]Biomedical Engineering Research Center, Smart Healthcare Research Institute, Samsung Medical Center, Seoul, Korea

[2]Department of Medical Device Management and Research, Samsung Advanced Institute for Health Sciences & Technology, Sungkyunkwan University, Seoul, Korea

[3]Center for Clinical Medicine, Samsung Biomedical Research Institute, Samsung Medical Center, Seoul, Korea

[4]Department of Laboratory Medicine and Genetics, Samsung Medical Center, Sungkyunkwan University School of Medicine, Seoul, South Korea

[5]Department of Medical Device Management and Research, Samsung Advanced Institute for Health Sciences & Technology, Sungkyunkwan University, Seoul, Korea.

[¶]These authors contributed equally to this work.

*** Corresponding authors**

Tae Yeul Kim; Tel.: +82-2-3410-0978; Fax: +82-2-3410-2719; E-mail address: voltaire0925@gmail.com.

Hee Jae Huh; Tel.: +82-2-3410-1836; Fax: +82-2-3410-2719; E-mail address: pmhhj77@gmail.com

Table S1. Optimal extraction-free protocols and their corresponding sensitivities described in previous studies and this study.

	Freppel, W. et al[1]	Fomsgaard, A. S. et al[2]	Genoud, V. et al.[3]	Villota, S. D. et al.[4]	Mallmann, L. et al. [5]	This study
rRT-PCR kit	Allplex™ 2019-nCoV Assay (Seegene Inc.)	In-house rRT-PCR	DisCoVery SARS-CoV-2 RT-PCR Detection Kit (Safecare Biotech Hangzhou Co. Ltd.)	Group 1: Detection Kit for 2019-nCoV (PCR-Fluorescence) (DaAnGene Co. Ltd.)<br>Group 2, 3, 4: In-house rRT-PCR	In-house rRT-PCR	STANDARD™ M SARS-CoV-2 Real-Time Detection Kit (SD Biosensor Inc.)
No. of positive samples	90	38	106	Group 1: 23<br>Group 2: 100<br>Group 3: 20<br>Group 4: 19	71	120
Range of Ct values	15 to 40 (mean Ct: 29.7)	N/A	N/A	Group 1: N/A<br>Group 2: 17.0 to 29.8* (n = 50)<br>30.1 to 37.8* (n = 50)<br>Group 3: N/A<br>Group 4: 22.0 to 36.2*	N/A	8.0 to 37.7 (mean Ct: 22.7)
Dilution	1/2 dilution	No	No	No	No	1/2 dilution
Add RNase inhibitor	No	No	No	Yes	No	Yes
Add Proteinase K	Yes	No	Yes	No	Yes	Yes
Heat treatment	Yes	Yes	Yes	No	Yes	Yes
Sensitivity (%)	98.9	97.4	99.0	100 in all four groups	94.4	84.3

N/A, not available; Ct, cycle threshold

*: Mean Ct value was not available.

Table S2. Comprehensive overview of all experimental conditions.

**Experimental condition**	**I**	**II**	**III**	**IV**	**V**	**VI**
RNA extraction	Y	N	N	N	N	N
1/2 dilution in RNase-free water	N	N	Y	Y	Y	Y
Add Proteinase K	N	N	N	N	Y	Y
Add RNase inhibitor	N	N	N	Y	N	Y
Heat treatment prior to the RT-PCR	N	98 °C for 5 min<br>4 °C for 2 min	98 °C for 5 min<br>4 °C for 2 min	55 °C for 15 min<br>98 °C for 5 min<br>4 °C for 2 min	98 °C for 5 min<br>4 °C for 2 min	55 °C for 15 min<br>98 °C for 5 min<br>4 °C for 2 min

I, standard methodology using the extraction step; II, extraction-free protocol based on heat treatment; III, extraction-free protocol based on heat treatment and sample dilution; IV, extraction-free protocol based on heat treatment, sample dilution, and addition of Proteinase K; V, extraction-free protocol based on heat treatment, sample dilution, and addition of RNase inhibitors; VI, extraction-free protocol based on heat treatment, sample dilution, and addition of Proteinase K and RNase inhibitors.

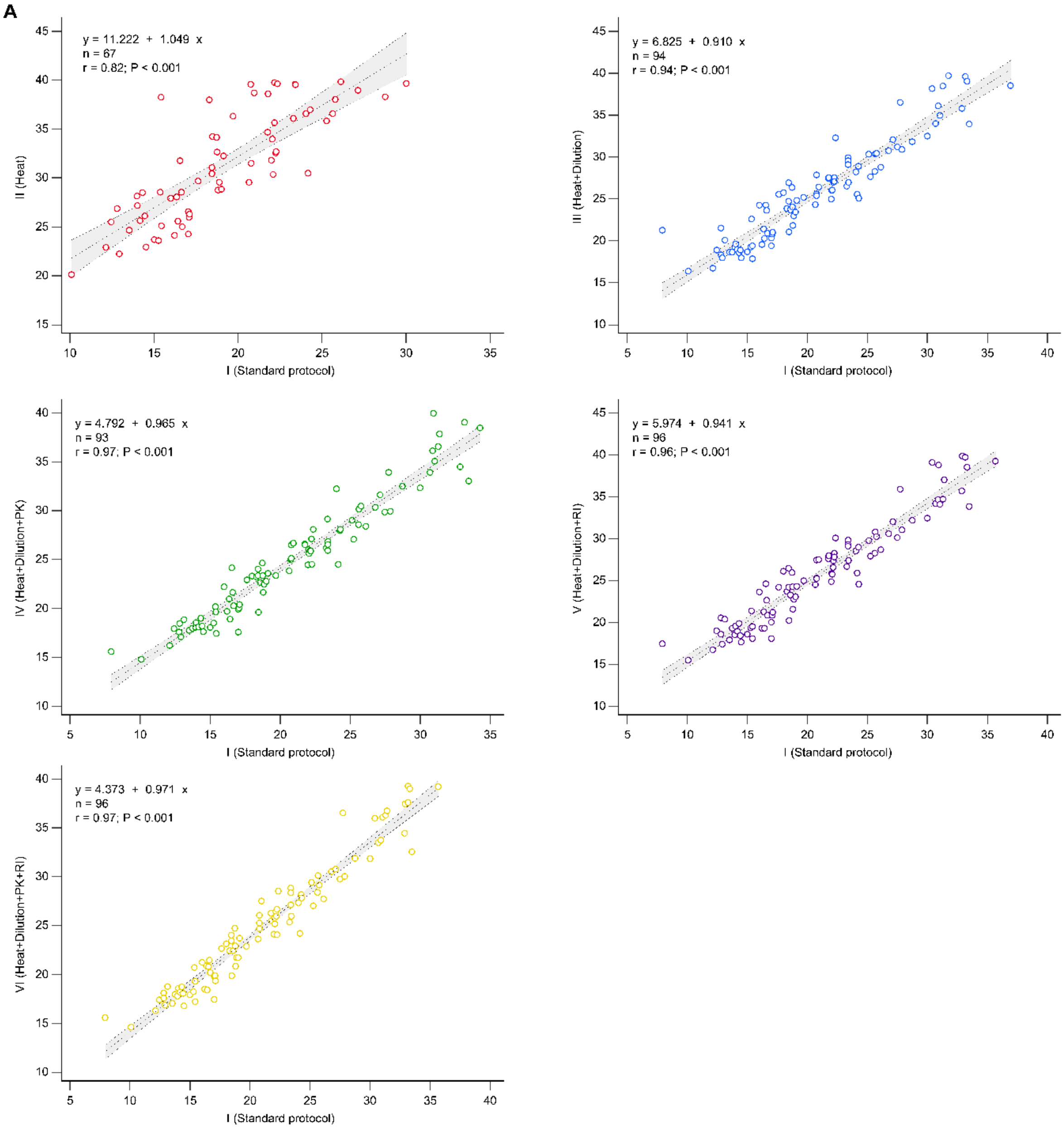
A
y = 11.222 + 1.049 x
n = 67
r = 0.82; P < 0.001
II (Heat)
I (Standard protocol)
y = 6.825 + 0.910 x
n = 94
r = 0.94; P < 0.001
III (Heat+Dilution)
I (Standard protocol)
y = 4.792 + 0.965 x
n = 93
r = 0.97; P < 0.001
IV (Heat+Dilution+PK)
I (Standard protocol)
y = 5.974 + 0.941 x
n = 96
r = 0.96; P < 0.001
V (Heat+Dilution+RI)
I (Standard protocol)
y = 4.373 + 0.971 x
n = 96
r = 0.97; P < 0.001
VI (Heat+Dilution+PK+RI)
I (Standard protocol)

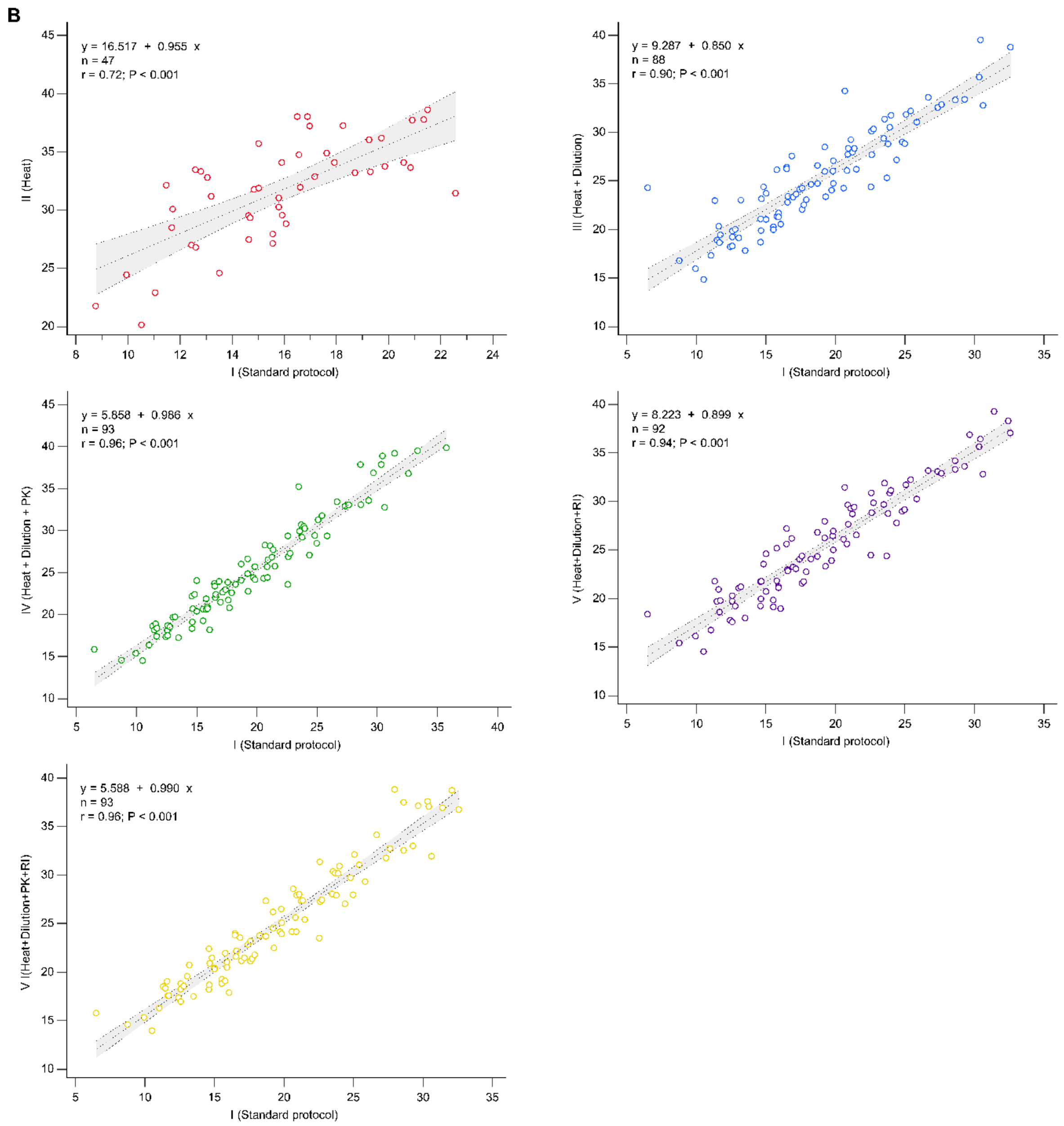

Figure S1. Comparison between Ct values of each target gene obtained using the standard protocol (x-axis) and the extraction-free protocols (y-axis) for (A) the ORF1ab and (b) the N gene. The solid blue line represents the regression analysis of the data, while the gray-shaded area represents the 95% confidence bands.

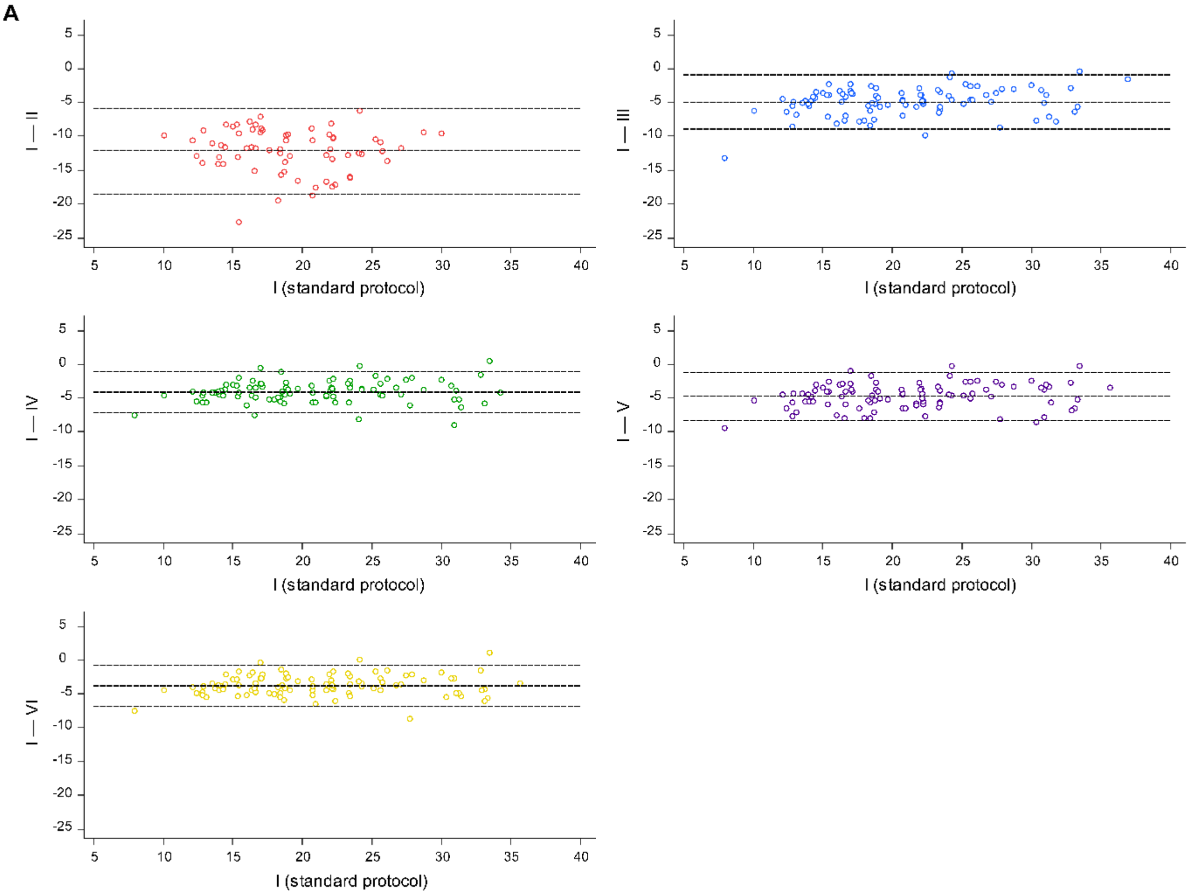
A
I — II
I — III
I — IV
I — V
I — VI
I (standard protocol)
I (standard protocol)
I (standard protocol)
I (standard protocol)
I (standard protocol)

B

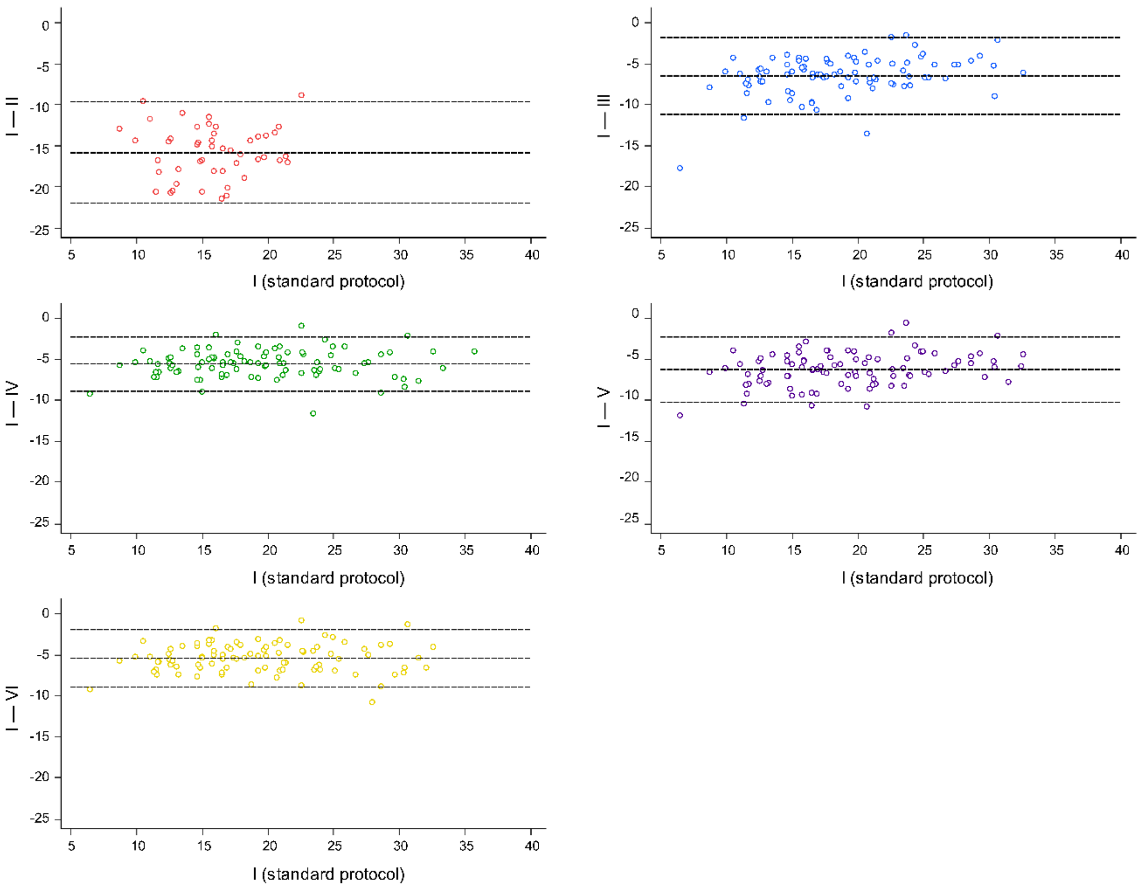

Figure S2. Bland-Altman plot for the differences between the standard protocol (x-axis) and the extraction-free protocols (y-axis). The blue line represents the mean difference of each group, while the red dotted lines depict the range within the 95% limits of agreement. (I, standard methodology using the extraction step; II, extraction-free protocol based on heat treatment; III, extraction-free protocol based on heat treatment and sample dilution; IV, extraction-free protocol based on heat treatment and sample dilution, and addition of Proteinase K; V, extraction-free protocol based on heat treatment and sample dilution, and addition of RNase inhibitors; VI, extraction-free protocol based on heat treatment, sample dilution, and addition of Proteinase K and RNase inhibitors)

Table S3. Details of 22 specimens showing discordant results between standard methodology using the extraction step and extraction-free protocol within the group that incorporated sample dilution (III-VI)

No.	Specimen No.	I	II	III	IV	V	VI
1	1	+	-	-	-	-	-
2	2	+	-	-	-	-	-
3	5	+	-	-	-	-	-
4	10	+	-	-	-	-	-
5	12	+	-	-	-	-	-
6	27	+	-	-	+	+	+
7	33	+	-	-	-	-	+
8	65	+	-	-	-	-	-
9	71	+	-	-	-	-	-
10	83	+	-	-	-	-	-
11	90	+	-	-	-	-	-
12	92	+	-	-	-	-	-
13	93	+	-	-	+	+	+
14	97	+	-	-	-	-	-
15	100	+	-	-	-	+	+
16	109	+	-	-	-	-	-
17	111	+	-	-	-	-	-
18	117	+	-	-	-	-	-
19	121	+	-	-	-	-	-
20	130	+	-	-	-	-	-
21	149	+	-	-	-	-	-
22	151	+	-	-	+	+	+

I, standard methodology using the extraction step; II, extraction-free protocol based on heat treatment; III, extraction-free protocol based on heat treatment and sample dilution; IV, extraction-free protocol based on heat treatment, sample dilution, and addition of Proteinase K; V, extraction-free protocol based on heat treatment, sample dilution, and addition of RNase inhibitors; VI, extraction-free protocol based on heat treatment, sample dilution, and addition of Proteinase K and RNase inhibitors.

1 Freppel, W., Merindol, N., Rallu, F. & Bergevin, M. Efficient SARS-CoV-2 detection in unextracted oro-nasopharyngeal specimens by rRT-PCR with the Seegene Allplex 2019-nCoV assay. *Virol J* **17**, 196, doi:10.1186/s12985-020-01468-x (2020).

2 Fomsgaard, A. S. & Rosenstierne, M. W. An alternative workflow for molecular detection of SARS-CoV-2 – escape from the NA extraction kit-shortage, Copenhagen, Denmark, March 2020. *Eurosurveillance* **25**, 2000398, doi:doi:https://doi.org/10.2807/1560-7917.ES.2020.25.14.2000398 (2020).

3 Genoud, V. *et al.* Extraction-free protocol combining proteinase K and heat inactivation for detection of SARS-CoV-2 by RT-qPCR. *PLoS One* **16**, e0247792, doi:10.1371/journal.pone.0247792 (2021).

4 Villota, S. D. *et al.* Alternative RNA extraction-free techniques for the real-time RT-PCR detection of SARS-CoV-2 in nasopharyngeal swab and sputum samples. *Journal of Virological Methods* **298**, 114302, doi:https://doi.org/10.1016/j.jviromet.2021.114302 (2021).

5 Mallmann, L. *et al.* Proteinase K treatment in absence of RNA isolation classical procedures is a quick and cheaper alternative for SARS-CoV-2 molecular detection. *Journal of Virological Methods* **293**, 114131, doi:https://doi.org/10.1016/j.jviromet.2021.114131 (2021).
